# Evaluation of Nebulized Tranexamic Acid for Acute Secondary Posttonsillectomy Hemorrhage

**DOI:** 10.1002/oto2.70296

**Published:** 2026-08-27

**Authors:** Chloe M. Jones, Donald H. Solomon, Bailey Balouch, Lucia D. Garcia, Yekaterina Shapiro

**Affiliations:** ^1^ Cooper Medical School of Rowan University Camden New Jersey USA; ^2^ Division of Otolaryngology ‐ Head and Neck Surgery Cooper University Hospital Camden New Jersey USA; ^3^ MD Anderson Cancer Center at Cooper Camden New Jersey USA

**Keywords:** control of oropharyngeal hemorrhage, nebulized tranexamic acid, postoperative bleeding, posttonsillectomy hemorrhage, TXA

## Abstract

**Objectives:**

Determine the efficacy of nebulized TXA for secondary posttonsillectomy hemorrhage.

**Study Design:**

Retrospective cohort study.

**Setting:**

Tertiary care medical center.

**Methods:**

All adult and pediatric patients who presented with active posttonsillectomy hemorrhage >24 hours posttonsillectomy treated at our tertiary care institution between December 2014 and July 2025 were included. Patients who were not actively bleeding on presentation were excluded. Outcomes were compared between patients who received nebulized TXA and those who did not. Outcomes included need for operative intervention, length of hospital stay, and recurrent hemorrhage rate. Univariate statistical analysis was performed using SPSS.

**Results:**

There were 101 patients included and 57 received nebulized TXA. All included patients were actively bleeding at the time of initial presentation. Hemostasis was achieved in 84.2% of the 57 patients following administration of nebulized TXA. Patients who received nebulized TXA were significantly less likely to require operative intervention (OR = 0.370 [0.157‐0.873]). If operative intervention was required following administration of nebulized TXA (54.9%), the time from presentation to operative intervention was significantly longer (408 ± 324 minutes vs 249 ± 293 minutes, *P* = .045). Neither hospital length of stay (1.2 ± 1.1 vs 1.5 ± 1.4 days, *P* = .148) or readmission rate for recurrent posttonsillectomy hemorrhage (7.0% vs 13.6%, *P* = .283) differed significantly between groups. No adverse effects of nebulized TXA were identified.

**Conclusion:**

Administration of nebulized TXA is a safe and effective strategy for acute stabilization of secondary posttonsillectomy hemorrhage. Nebulized TXA reduced the rate of operative intervention for secondary posttonsillectomy hemorrhage and increased the time until intervention was required.

Tonsillectomy is one of the most common surgeries performed in the United States, with over 530,000 procedures for pediatric and adult patients per year.^1^ The most common indications for tonsillectomy include obstructive sleep apnea and recurrent tonsillitis.^2^ Postoperative complications include nausea, vomiting, pain, dehydration, and bleeding.^3^ Posttonsillectomy hemorrhage is a well‐known complication following tonsillectomy, occurring in up to 5% of pediatric patients and 13.6% of adult patients.^4^ Primary posttonsillectomy hemorrhage occurs within 24 hours after surgery, and is generally thought to be a result of technical error, while secondary hemorrhage occurs after the first 24 hours postoperatively.^5^ While many hemorrhages resolve without medical or surgical intervention, approximately 50% will require operative intervention.^6^ Conservative interventions to control hemorrhage at the bedside range from ice water gargles, application of direct pressure, use of silver nitrate or electrical cautery, or administration of topical vasoconstrictors such as epinephrine.^5^ While these interventions may help to stop bleeding, they are limited by patient cooperation and access to the site of bleeding, and none have been proven to reduce the need for operative intervention.^7^, ^8^ Operative intervention includes removal of any clot that has formed in the tonsillar fossae, identifying the source of hemorrhage, and performing cauterization to achieve definitive hemostasis, typically done it the operating room under general anesthesia.

Tranexamic acid (TXA) treatment has become a more frequent intervention for posttonsillectomy hemorrhage in recent years. TXA is a lysine analog that competitively inhibits the conversion of plasminogen to plasmin, preventing the degradation of fibrin clots and reducing bleeding.^4^ In addition, by inhibiting plasmin activation it exhibits anti‐inflammatory properties by preventing plasmin‐mediated complement and inflammatory cell activation.^7^ TXA is FDA‐approved for intravenous treatment of traumatic and postpartum hemorrhage.^1^ In 2019, Schwarz et al first described the use of nebulized TXA to achieve hemostatic control after posttonsillectomy bleeding failed to cease, allowing time for definitive operative management.^8^ Erwin et al showed that nebulized TXA decreased the need for operative management in a series of 14 patients from 73% to 29% compared to controls.^9^ Maksimiski et al reported that no patients required operative intervention in a series of 6 patients who received nebulized TXA compared to 47% of controls. Spencer et al found that 22% of 17 TXA‐treated versus 54% of controls required operative cautery, though TXA was applied via nebulizer, topically, or intravenously in that study.^4^ Ojeaga et al demonstrated a reduction in operative intervention from 41.7% in controls to 26.0% in 100 patients treated with nebulized TXA.^10^ Alghamdi et al conducted a meta‐analysis of 136 patients that showed that patients treated with TXA had a significantly lower rate of surgical intervention to control bleeding, with no reports of medication side effects such as thromboembolic events.^7^ While these prior studies have shown success with nebulized TXA for treatment of posttonsillectomy hemorrhage, they have been limited in sample size and varied with regards to inclusion criteria and TXA dosing. The purpose of this study was to determine the efficacy of nebulized TXA in patients presenting with active secondary posttonsillectomy hemorrhage at our institution.

## Methods

This study was approved by the Cooper University Hospital Institutional Review Board (Protocol #24‐312). This was a retrospective study of adult and pediatric patients who presented to our tertiary care center for secondary posttonsillectomy hemorrhage between December 2014 and July 2025. There were approximately 3882 tonsillectomies performed at our institution over the study period (2681 pediatric and 1201 adult). Patients who presented with postoperative bleeding less than 24 hours after surgery and patients who were not actively bleeding on presentation were excluded from this study. All tonsillectomies were performed with extracapsular dissection using coblator, bipolar scissors, or monopolar electrocautery. At our institution, nebulized TXA was administered as a standardized dose of 500 mg (5 mL of 100 mg/mL intravenous TXA solution) via nebulizer. Patients typically received a single dose of nebulized TXA. If additional doses were necessary, an additional 500 mg of TXA was administered via nebulizer per dose. Dosage was not altered for pediatric patients. The first time nebulized TXA was used at the study institution was in April of 2019 and routine use of nebulized TXA for posttonsillectomy hemorrhage was adopted by June of 2020. Outcomes including the need for operative intervention, length of hospital stay, and rate of recurrent posttonsillectomy hemorrhage requiring a repeat emergency department and/or inpatient encounter were compared between patients who received nebulized TXA and those who did not. Complete versus temporary hemostasis was not able to be obtained from the medical records.

Statistical analysis was performed using SPSS version 29.0.2.0 (IBM). Descriptive statistics were used to summarize baseline patient characteristics. Chi‐square or Fisher's exact test was used to evaluate differences between groups in nominal scale variables. Independent samples *t*‐test was used to evaluate differences between groups in continuous variables. Subgroup analysis comparing differences between multiple groups was achieved using the Fisher‐Freeman‐Halton exact test for nominal variables and one‐way analysis of variance (ANOVA) for continuous variables, with post hoc Games‐Howell test to be used when appropriate. All counts are presented as percentages and means are presented as the mean ± standard deviation (SD). All tests were performed 2‐tailed and a *P* < .05 was considered statistically significant.

## Results

101 patient encounters for actively bleeding secondary posttonsillectomy hemorrhage were included in this study. The average age was 21.5 ± 11.7 (range = 3‐61). There were 37 pediatric patients (ages 3‐17) and 64 adult patients (ages 18‐61). The estimated rate of posttonsillectomy hemorrhage was 2.6% (1.4% for pediatrics and 5.3% for adults). There were 55 males (54.5%) and 46 females (45.5%) included in this study. Indications for tonsillectomy included recurrent or chronic tonsillitis (54.5%), tonsillar hypertrophy (40.6%), OSA (34.7%), and tonsilloliths (1.0%). The average postoperative day when patients presented with hemorrhage was 7.2 ± 3.3 (range = 1‐17 days posttonsillectomy).

Nebulized TXA was administered to 57 patients (56.4%) and at least temporary hemostasis was achieved with nebulized TXA in 48 (84.2%). On average, 1.1 ± 0.5 doses of nebulized TXA were administered (1 dose: n = 47, 2 doses: n = 9, 3 doses: n = 1). Forty‐four patients did not receive TXA. Of the patients in the control group who did not receive nebulized TXA, 29 were in the early cohort prior to routine use of nebulized TXA and 15 were in the late cohort after use of nebulized TXA had been adopted by the institution. Sixty‐three patients (62.4%) required operative intervention for control of hemorrhage, 30 from the nebulized TXA group and 33 from the non‐TXA group. Patients who received nebulized TXA were significantly less likely to require operative intervention for posttonsillectomy hemorrhage compared to those who did not receive nebulized TXA (52.6% vs 75.0%, OR = 0.370 [0.157‐0.873], *P* = .024). When stratified by patient age, a similar trend was observed in the pediatric (44.4% vs 68.4%, OR = 0.369 [0.097‐1.412], *P* = .191) and adult subsets (56.4% versus 80.0%, OR = 0.324 [0.101‐1.039], *P* = .064) but neither were significant. Administration of nebulized TXA was also associated significantly with a longer duration from time of presentation with hemorrhage to need for operative intervention (408 ± 324 vs 249 ± 293 minutes, *P* = .045). Neither operative time (22.2 ± 11.2 vs 17.6 ± 13. 8 minutes, *P* = .172) nor anesthesia time (45.2 ± 14.7 vs 39.2 ± 16.5, *P* = .154) differed significantly between those who received nebulized TXA and those who did not (Table 1).

**Table 1:** Baseline Patient Characteristics and Outcomes After Secondary Posttonsillectomy Hemorrhage

	Nebulized TXA	No TXA	Difference	Sig. (*P*‐value)
Total number (n)	57	44	‐	‐
Age (mean ± SD)	22.8 ± 12.4	19.9 ± 10.8	2.868 [−1.711 to 7.447]	.225
Male gender (n [%])	32 [56.1%]	23 [52.3%]	0.039 [−0.154 to 0.230]	.699
Recurrent or chronic tonsillitis (n [%])	34 [59.7%]	21 [47.7%]	0.119 [−0.076 to 0.306]	.233
Tonsillar hypertrophy (n [%])	19 [33.3%]	22 [50.0%]	−0.167 [−0.349 to 0.027]	.091
Obstructive Sleep Apnea (n [%])	18 [31.6%]	17 [38.6%]	−0.071 [−0.254 to 0.115]	.460
Number of days between tonsillectomy and hemorrhage (mean ± SD)	7.0 ± 3.5	7.6 ± 3.0	−0.576 [−1.899 to 0.747]	.390
Hemoglobin level in g/dL (mean ± SD)	12.6 ± 1.7	12.3 ± 2.1	0.299 [−0.470 to 1.067]	.442
Platelets per mcL (mean ± SD)	313.6 ± 67.7	331.3 ± 91.2	−17.682 [−49.721 to 14.358]	.276
Need for red blood cell transfusion (mean ± SD)	5 [8.8%]	2 [4.6%]	0.042 [−0.069 to 0.142]	.465
Need for operative control of hemorrhage (n [%])	30 [52.6%]	33 [75.0%]	−0.224 [−0.394 to −0.034]	.024
Time from presentation to operating room in minutes (mean ± SD)	408 ± 324	249 ± 293	159.23 [3.851 to 314.609	.045
Operative time in minutes (mean ± SD)	22 ± 11	18 ± 14	4.579 [−2.055 to 11.212]	.172
Anesthesia time in minutes (mean ± SD)	45 ± 15	39 ± 17	5.945 [−2.296 to 14.187]	.154
Hospital length of stay in days (mean ± SD)	1.2 ± 1.1	1.5 ± 1.4	−0.362 [−0.854 to 0.130]	.148
Recurrent posttonsillectomy hemorrhage (n [%])	4 [7.0%]	6 [13.6%]	−0.065 [−0.192 to 0.060]	.283

Continuous variables are presented as the (mean ± SD). Statistical differences were assessed using the independent samples *t*‐test. Differences between means with corresponding 95% confidence interval are provided.

Nominal variables are presented as counts with corresponding percentages. Statistical differences were assessed using the Chi‐Square test. Differences in proportions with corresponding 95% confidence intervals are provided.

*P *< .05 were considered statistically significant.

Hospital length of stay did not differ significantly (1.2 ± 1.1 vs 1.5 ± 1.4 days, *P* = .148) between those who received nebulized TXA and those who did not (Table 1). The rate of recurrent posttonsillectomy hemorrhage resulting in a repeat emergency department encounter also did not differ significantly (7.0% vs 13.6%, *P* = .283) between those who received nebulized TXA and those who did not (Table 1). Repeat admission or emergency department encounter occurred an average of 2.2 ± 2.1 days following discharge from the initial encounter for posttonsillectomy hemorrhage (range = 0‐6 days).

**Figure 1:**
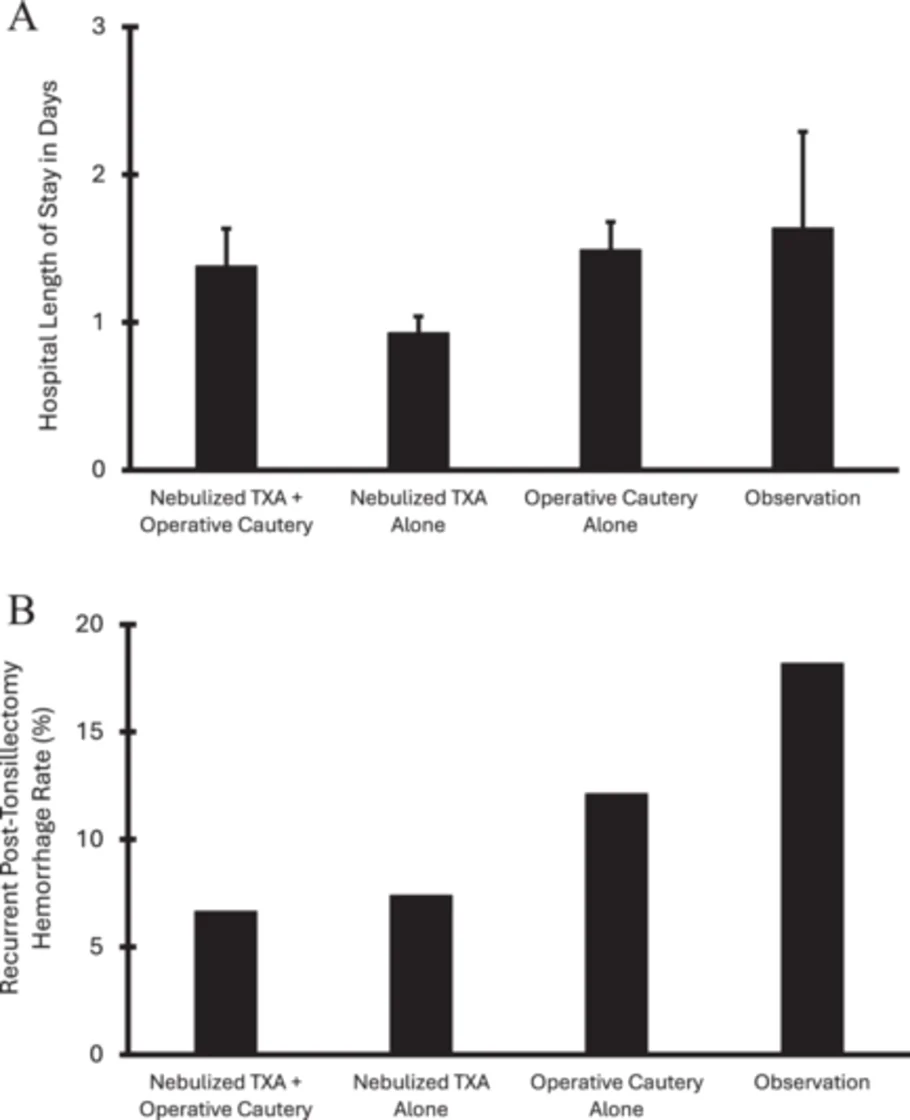
Subgroup analysis stratified by treatment modalities delivered. (A) Hospital length of stay (in days) between groups. Error bars represent standard error of the mean. (B) Recurrent posttonsillectomy hemorrhage (rebleed) rate between groups.

Secondary posttonsillectomy hemorrhage patients were further subgrouped based on treatment modalities utilized: Dual treatment with nebulized TXA and operative cautery (n = 30), nebulized TXA alone (n = 27), operative cautery alone (n = 33), or observation (n = 11). Hospital length of stay did not differ significantly between groups (*F* = 1.384, *P* = .252, see Figure 1A). The rate of recurrent posttonsillectomy hemorrhage also did not differ significantly between groups (*P* = .630, see Figure 1B). No adverse effects of nebulized TXA were observed in this study.

## Discussion

The goal of this study was to determine the safety and efficacy of nebulized TXA administration for secondary posttonsillectomy hemorrhage. In our study, consistent with what has been reported in the literature, administration of nebulized TXA significantly reduced the need for operative cautery in secondary posttonsillectomy hemorrhage. Similarly, in patients that required operative intervention, administration of TXA safely delayed time to operation without impacting operative time or overall length of hospital stay. No adverse effects associated with the use of nebulized TXA were identified. These findings support the use of nebulized TXA as a management strategy for posttonsillectomy hemorrhage or as a temporizing measure to achieve hemostasis as a bridge to definitive operative intervention.

TXA nebulization achieved hemostasis in 84% (48/57) of patients in this study, which is comparable to the current literature.^9^ Achieving early hemostasis in posttonsillectomy hemorrhage reduces the risk for airway compromise, such as with aspiration, obstruction, or laryngospasm. Posttonsillectomy hemorrhage remains the leading cause of posttonsillectomy mortality, most frequently in pediatric populations. Earlier cessation of active posttonsillectomy hemorrhage leads to decreased blood loss and need for transfusion which may improve hemodynamic stability hence could improve rates of shock or mortality.^11^ By reducing the incidence of operative intervention, nebulized TXA treatment also limits a secondary anesthetic exposure and the risk for operative complications. Further, while TXA nebulization was not shown to reduce overall length of hospital stay, eliminating operative intervention corresponds to lower healthcare costs overall.

Comparison across current literature is limited by varied study inclusion criteria; while our study and Spencer et al^4^ were specific for cases of secondary posttonsillectomy hemorrhage with active bleeding at the time of presentation, both Erwin^9^ and Shin et al^12^ included cases of both primary and secondary posttonsillectomy hemorrhage. Ojeaga et al included all patients who experienced posttonsillectomy bleeding, regardless of whether active bleeding was present at the time of evaluation,^10^ and other studies often do not specify. Larger scale or multicenter studies may provide greater generalizability. In addition, current studies report nebulized TXA efficacy with varied protocols. The majority of patients received a single dose of nebulized TXA in our study, with a minority of patients receiving 2 doses (n = 9) or 3 doses (n = 1) based on the clinical judgement of the physician. Whereas Erwin et al administered a standardized three doses per pediatric patient,^9^ while Spencer et al based dosing on patient weight.^4^

Further research should aim to optimize a dose delivery protocol. Further, current protocols compare TXA with varied control measures depending on institutional preference, such as conservative ice water gargles, silver nitrate cautery, or a mixture of bedside interventions. These control methods should be standardized in order to effectively compare with TXA nebulization. The combination of nebulized TXA with measures like silver nitrate may be explored for possible synergism.

Our study was limited by its retrospective nature and sample size. The indication for secondary doses of TXA was unable to be obtained from the chart, which may reflect either regarding persistent or recurrent bleeding. Further, outcomes did not include whether patients were found to be coagulopathic, which may have contributed to prolonged bleeding. However, to our knowledge this is one of the largest retrospective cohort studies to date to investigate the efficacy of nebulized TXA for control of secondary posttonsillectomy hemorrhage in actively bleeding patients. Our study sample was limited to those actively bleeding at presentation, while those who achieved spontaneous resolution of hemorrhage prior to evaluation were excluded. This strict inclusion criteria allowed for us to investigate the efficacy of nebulized TXA just in those patients who in practice it would be most useful.

Additionally, in many cases operative intervention was performed in patients who had achieved hemostasis with nebulized TXA to remove clot and localize the source of bleeding. At our institution, patients who are hemostatic with clot present in the fossa may be taken to the operating room for clot disruption and cautery or challenged with a liquid diet to disrupt the clot while in a monitored setting for 24 hours, depending on provider preference. The number of patients who ultimately underwent operative intervention after receiving nebulized TXA may therefore have been inflated by inclusion of patients who underwent operative intervention despite having already achieved hemostasis with nebulized TXA. There was no difference in recurrent hemorrhage rate between those who received nebulized TXA followed by operative cautery compared to those who received nebulized TXA alone.

## Conclusion

Administration of nebulized TXA is a non‐invasive, safe, and effective strategy for acute stabilization of secondary posttonsillectomy hemorrhage. Nebulized TXA reduces the rate of operative intervention for posttonsillectomy hemorrhage as well as allows operative intervention to be safely delayed when necessary. Readmission rates and length of stay are similar between those patients who receive TXA and those who do not. No adverse effects of nebulized TXA were observed in this study.

## Author Contributions

**Chloe M. Jones**, conduct, presentation; **Donald H. Solomon**, analysis, conduct; **Bailey Balouch**, design, analysis, presentation; **Lucia D. Garcia**, analysis; **Yekaterina Shapiro**, supervision, design, analysis, presentation.

## Disclosures

### Competing interests

None.

### Funding source

None.
